# Sex related difference in postoperative pain and opioid use following posterior spinal fusion for adolescent idiopathic scoliosis

**DOI:** 10.1007/s43390-024-00826-x

**Published:** 2024-02-08

**Authors:** Reid W. Collis, Tonia Dry, Gilbert Chan, Poh Lim, Timothy Oswald

**Affiliations:** 1 Department of Physical Medicine and Rehabilitation, University of Virginia School of Medicine, 545 Ray C. Hunt Drive, Suite #2400, P.O. Box #801004, Charlottesville, VA 22903 USA; 2 Department of Pediatric Orthopedics, Wellstar Health System, Marietta, GA USA

**Keywords:** Adolescent, Idiopathic, Scoliosis, Fusion, Opioid, Pain

## Abstract

**Purpose:**

This project aims to evaluate the relationship between biological sex and postoperative pain and in patients receiving posterior spinal fusion for adolescent idiopathic scoliosis.

**Methods:**

This is a retrospective study of patients (n=137) aged 10-17 receiving posterior spinal fusion for adolescent idiopathic scoliosis between 01/2018 and 09/2022. Each patient received surgery by the same pediatric orthopedic surgeon with identical postoperative pain management regimen at a children’s hospital or a tertiary referral center with a pediatric spine program.

**Results:**

There were no significant differences in any background characteristics between the male and female patients, including age, BMI, number of levels fused, preoperative degree of scoliosis, and length of surgery and anesthesia (p>0.05). There were no differences in amount given of any intraoperative medications, nor in amount of postoperative scheduled analgesics (p>0.05). Female patients demonstrated higher average pain scores on Visual Analogue Scale evaluations during the first 24 hours postoperatively (5.0 vs 3.6, p<0.0001), 24-48 hours postoperatively (4.9 vs 4.0, p=0.03), and at the first physical therapy evaluation (5.3 vs 3.8, p<0.001). These patients received significantly greater amounts of morphine milligram equivalents in the first 24 hours postoperatively (42.2 vs 31.5, p=0.01) and for the hospitalization in total (63.8 vs 51.3, p=0.048). There was no difference in hours until hospital discharge (44.3 vs 42.6, p=0.62) nor until first ambulation (20.1 vs 21.3, p=0.24) between the female and male patients.

**Conclusion:**

The influence of biopsychosocial factors on postoperative pain in adolescents is complex. This study adds to the existing pool of literature suggesting differences in pain perception between adolescent female and male patients. Female patients undergoing posterior spinal fusion for adolescent idiopathic scoliosis may benefit from increased preoperative counseling and more aggressive intra- and postoperative pain management regimens.

## Introduction

Adolescent idiopathic scoliosis (AIS) is a condition found in otherwise healthy adolescents in approximately 1–4% of the population and is defined as a lateral curvature of the spine greater than 10 degrees, as measured by the Cobb angle, accompanied by vertebral rotation [1, 2]. While the ratio of females to males with spinal curvature of 10 degrees is equal, greater degrees of curvature are found more commonly in females than males, particularly with curvature greater than 30 degrees. At these levels, the ratio of females to males is typically 10-to-1 [3]. There are many variables that factor into the decision to pursue surgical correction versus conservative management; however, curves exceeding 40–50 degrees are usually treated with surgical intervention. In patients requiring surgical correction, posterior spinal fusion (PSF) is a commonly performed surgery involving the fusion and realignment of the vertebrae to correct the underlying deformity.

Posterior spinal fusion involves significant tissue trauma and is associated with debilitating pain [4]. Historically, opioid medications have been the mainstay of management of postoperative pain [5, 6]. With ever expanding literature reinforcing the risks associated with post operative opioid use, namely increasing risk for persistent opioid use and overdose, research into alternative methods of reduction of postoperative pain and opioid use has become a focus within the field of pediatric orthopedics [5–7]. Novel methods of intra- and perioperative pain control, including use of multimodal pain management regimens and intraoperative liposomal bupivacaine administered subcutaneously into the lumbar and thoracic paraspinal musculature just prior to PSF, have proven efficacious in reduction of postoperative pain and hospital length of stay following spine surgery [8–11].

The differential expression and subjective experience of postoperative pain between males and females is a well-studied and complex topic, situated squarely at the intersection between biological and psychosocial influences. Research in this field indicates that males and females tend to experience and respond to pain differently. In adult populations, some clinical studies suggest females have lower pain thresholds and less pain tolerance compared to males [12] and may report greater pain after surgery [13], though adult males appear to require higher doses of opioid analgesics than their female counterparts postoperatively [14]. In the pediatric population, however, there is even less of a clear demarcation in inter-sex differences in subjective pain experience. Furthermore, given the increased complexity of biopsychosocial influences in this particular population, ascertaining the etiologies underlying differential expression of pain between sexes remains challenging. There is a relatively small body of literature regarding differences in postoperative pain as it relates to biologic sex in the adolescent population. Studies suggest that differences in anticipatory distress prior to surgery between adolescent girls and boys may be associated with differences in reports of postoperative pain [15, 16], though a clear relationship between sex and degree of subjective pain postoperatively has not been demonstrated [16, 17].

The goal of this retrospective study is to evaluate patient outcomes including postoperative pain control and analgesic use between female and male patients following posterior spinal fusion for adolescent idiopathic scoliosis.

## Materials and methods

This is a retrospective analysis of adolescent girls (n = 107) and boys (n = 30) aged 10–17 who received posterior spinal fusion for adolescent idiopathic scoliosis between January 2018 and September 2022. Patients received surgery with the same pediatric orthopedic surgeon (TO) at one of two medical centers—a dedicated children’s hospital and a regional tertiary referral center with a dedicated pediatric spine program. Patients treated at each location did not differ with respect to preoperative evaluation, intraoperative surgical technique, or postoperative pain management protocols. Each patient received a morphine patient-controlled analgesia (PCA) pump with identical lockout and timing interval parameters, with discontinuation orders for postoperative day 1 (POD1) at 7:00am. Subsequent PRN opioids were prescribed with the same Visual Analogue Scale (VAS) pain score parameters. VAS scores were performed per physical therapy and nursing protocol at each patient interaction. Patients were excluded from evaluation if they did not meet age or date of surgery criteria, if they received or had previously received any spinal surgery or procedure other than or in addition to posterior spinal fusion, or if they presented with non-idiopathic scoliosis (e.g. syndromic scoliosis, neuromuscular scoliosis, congenital malformation, etc.). Categorical variables were expressed as counts and percentages. Continuous variables were expressed as means and standard deviations. Between group comparisons were performed with a chi square test for categorical variables and a Student’s t-test for continuous variables. A P-value less than 0.05 was considered to be statistically significant.

## Results

In evaluating the effect of hospital location, there was no difference in postoperative opioid use in patients receiving surgery at the children’s hospital versus the tertiary referral center when accounting for all other variables (p = 0.45). Patients receiving surgery at the children’s hospital did not differ from those receiving surgery at the tertiary referral center in background characteristics including age, female sex, height, weight, BMI, race, or preoperative cobb angle, nor in intraoperative characteristics including number of levels fused, estimated blood loss, or length of anesthesia (p > 0.05 for all comparisons).

Between the female and male patients, there similarly was no difference in each aforementioned background and intraoperative characteristic (p > 0.05 for all comparisons, Table 1). There additionally was no difference in the milligrams received of any intraoperative medication when comparing females versus males, including acetaminophen (523.5 vs 440.2, t = 1.0, df = 135, p = 0.34), non-liposomal bupivacaine (102.6 vs 80.8, t = 1.4, df = 135, p = 0.17), liposomal bupivacaine (172.5 vs. 137.4, t = 1.3, df = 135, p = 0.18), dexamethasone (6.7 vs. 5.7, t = 1.7, df = 135, p = 0.08), dexmedetomidine (1.7 vs 3.1, t = 1.1, df = 135, p = 0.30), diazepam (0.1 vs 0.2, t = 0.6, df = 135, p = 0.54), fentanyl (321.0 vs 340.7 (mcg), t = 0.5, df = 135, p = 0.61), hydromorphone (0.5 vs 0.4, t = 0.8, df = 135, p = 0.42), ketamine (29.8 vs 20.7, t = 1.8, df = 135, p = 0.08), ketorolac (17.1 vs 15.3, t = 0.7, df = 135, p = 0.48), lidocaine (38.4 vs 40.3, t = 0.3, df = 135, p = 0.78), methocarbamol (299.7 vs 224.2, t = 0.9, df = 135, p = 0.36), morphine (0.04 vs 0.3, t = 1.5, df = 135, p = 0.15), and remifentanil (140.0 vs 208.3 (mcg), t = 0.6, df = 135, p = 0.56) (Table 2). Female patients reported significantly higher postoperative pain ratings based on average visual analog scale (VAS) over the first 24 h after surgery (5.0 vs 3.6, t = 4.0, df = 135, p < 0.0001), 24-48 h after surgery (4.9 vs 4.0, t = 2.2, df = 101, p = 0.03), as well as at the first physical therapy evaluation (5.3 vs 3.8, t = 3.5, df = 127, p < 0.001) (Table 3). Female patients reported greater degrees of pain at the second physical therapy evaluation as well, though this difference did not meet statistical significance (4.9 vs 4.4, t = 0.9, df = 105, p = 0.37).

**Table 1:** Background characteristics of patients

Background characteristics	Female patients (n = 107)			Male patients (n = 30)			*p*
	Mean ± SD or n (%)		95% CI	Mean ± SD or n (%)		95% CI	
Age at surgery	14.2	± 1.7	14.05–14.41	14.7	± 1.3	14.43 to 14.90	0.23
Race	102			28			0.38
White	47	(46.1%)		14	(50.0%)		
Black	43	(42.2%)		10	(35.7%)		
Asian	1	(1.0%)		2	(7.1%)		
Hispanic/Latino	6	(4.9%)		1	(3.6%)		
Other	5	(5.9%)		1	(3.6%)		
Body mass index (BMI)	22.2	± 4.7	21.70–22.60	22.3	± 6.1	21.16–23.34	0.92
Surgery length (mins)	149.7	± 26.9	147.10–152.19	145.9	± 27.4	140.99–150.81	0.50
Anesthesia length (mins)	216.9	± 30.7	213.93–219.77	205.0	± 28.6	199.88–210.12	0.06
Number of levels fused	9.9	± 1.7	9.73–10.05	9.4	± 2.1	9.07–9.80	0.22
Cobb angle (degrees)	54.8	± 9.2	53.96–55.70	55.9	± 10.7	54.02–57.85	0.58

**Table 2:** Intraoperative medications given during posterior spinal fusion

Intraoperative medications*	Female patients (n = 107)			Male patients (n = 30)			Comparison		
	Mean ± SD	95% CI	N^†^	Mean ± SD	95% CI	N	Mean Diff	95% CI	*p*
*Acetaminophen*	523.5 ± 416.1	484.09–562.92	68	440.2 ± 429.1	363.39–516.94	16	83.3	− 87.81–254.49	0.34
*Bupivacaine (Non-liposomal)*	102.6 ± 76.4	95.38–109.85	43	80.8 ± 78.7	66.75–94.91	16	21.8	− 9.63–53.20	0.17
*Bupivacaine (Liposomal)*	172.5 ± 125.3	160.66–184.40	72	137.4 ± 132.9	113.65–161.22	16	35.1	− 16.79–86.97	0.18
*Dexamethasone*	6.7 ± 2.6	6.45–6.93	99	5.7 ± 3.4	5.09–6.31	23	1.0	− 0.13–2.12	0.08
*Dexmedetomidine (mcg)*	1.7 ± 6.2	1.08–2.26	9	3.1 ± 7.2	1.78–4.35	5	− 1.4	− 4.03–1.24	0.30
*Diazepam*	0.1 ± 0.6	0.03–0.14	3	0.2 ± 0.9	0.00–0.33	1	− 0.1	− 0.35–0.19	0.54
*Fentanyl (mcg)*	321.0 ± 186.8	303.29–338.68	102	340.7 ± 180.4	308.41–372.97	30	− 19.7	− 95.47–56.06	0.61
*Hydromorphone*	0.5 ± 0.5	0.43–0.53	74	0.4 ± 0.5	0.31–0.48	19	0.1	− 0.13–0.30	0.42
*Ketamine*	29.8 ± 25.1	27.39–32.14	67	20.7 ± 26.1	16.00–25.33	14	9.1	− 1.23–19.43	0.08
*Ketorolac*	17.1 ± 12.5	15.94–18.30	73	15.3 ± 13.5	12.85–17.68	30	1.8	− 3.32–7.04	0.48
*Lidocaine*	38.4 ± 32.8	35.26–41.47	70	40.3 ± 40.4	33.11–47.56	18	− 1.9	− 16.10–12.16	0.78
*Methocarbamol*	299.7 ± 404.7	261.39–338.07	40	224.2 ± 361.7	159.45–288.89	9	75.6	− 86.17–237.29	0.36
*Morphine*	0.04 ± 0.4	0.00–0.07	1	0.3 ± 1.5	0.01–0.53	1	− 0.2	− 0.54–0.08	0.15
*Remifentanil (mcg)*	140.0 ± 537.9	89.04–190.96	8	208.3 ± 676.2	87.35–329.32	3	− 68.3	− 301.40–164.74	0.56

*In milligrams unless otherwise noted

^†^Number of patients receiving each medication

**Table 3:** Postoperative characteristics of patients following posterior spinal fusion

Postoperative characteristics*	Female patients (n = 107)			Male patients (n = 30)			Comparison		
	Mean ± SD	95% CI	N	Mean ± SD	95% CI	N	Mean diff	95% CI	*p*
Average VAS									
First 24 h	5.0 ± 1.8	4.83–5.18	107	3.6 ± 0.5	3.37–3.88	30	1.4	0.70–2.07	** < 0.0001**
24 h-48 h	4.9 ± 1.9	4.67–5.08	77	4.0 ± 0.6	3.70–4.29	26	0.9	0.08–1.68	**0.03**
At 1st PT evaluation	5.3 ± 2.0	5.08–5.47	99	3.8 ± 0.6	3.54–4.15	28	1.5	0.63–2.23	** < 0.001**
At 2nd PT evaluation	4.9 ± 2.3	4.61–5.11	81	4.4 ± 0.9	3.92–4.83	23	0.5	− 0.58–1.56	0.37
First 24 h morphine milligram equivalents (mg)	42.2 ± 21.5	40.18–44.24	107	31.5 ± 17.6	28.34–34.64	30	10.7	2.26–19.17	**0.013**
Total postoperative morphine milligram equivalents (mg)	63.9 ± 30.6	60.95–66.75	107	51.3 ± 29.4	46.09–56.60	30	12.6	0.11–24.91	**0.048**
Hospital length of stay following surgery completion (h)	44.3 ± 17.7	42.64–45.99	107	42.6 ± 12.6	40.32–44.84	30	1.7	− 5.09–8.57	0.62
Discharge date									0.95
POD1^†^			43			11			
POD2			52			17			
POD3			8			2			
POD4			4			0			
Time until first recorded ambulation (h)	20.1 ± 5.4	19.61–20.63	107	21.3 ± 2.7	20.83–21.80	30	− 1.2	− 3.22–0.82	0.24

*All characteristics include the time period from the cessation of anesthesia to time of discharge unless otherwise noted

^†^POD = postoperative day, which refers to the number of calendar days following the date of surgery completion

Female patients received a greater number of postoperative morphine milligram equivalents in the first 24 h postoperatively (42.2 vs 31.5, t = 2.5, df = 135, p = 0.01) as well as during the duration of the hospitalization (63.9 vs 51.3, t = 2.0, df = 135, p = 0.048). There was no difference in hospital length of stay (44.3 h vs 42.6 h, t = 0.5, df = 135, p = 0.62) nor in time until first recorded ambulation (20.2 h vs 21.3 h, t = 1.2, df = 135, p = 0.24) between the two groups (Table 3). There was no difference in MME prescribed at hospital discharge (186.1 vs 185.1, t = 0.2, df = 135, p = 0.84). There were no all-cause 30-day readmissions for all patients.

## Discussion

In the adolescent population, a variety of biopsychosocial factors confound the determination of the degree and subjective experience of pain, particularly in circumstances such as the perioperative time period where heightened levels of anxiety and apprehension are common [18].

Prior studies in the adolescent population suggest that increased levels of anxiety and fear directly correlate with higher levels of pain catastrophization, independently contributing to the prediction of increased pain severity and depression [19, 20]. More specific to the perioperative time frame, existing research supports positive relationships between the expected degree of postoperative pain when assessed preoperatively and subsequent reported degree of pain in the postoperative period [21]. Additionally, emerging literature suggests direct impacts of sex steroid hormones on subjective pain perception in the peripubertal time period [22, 23]. This is particularly relevant to this patient population given the age range of patients included in this study overlaps significantly with the age range of puberty in both adolescent females and males [24].

In this patient cohort, females were found to have significantly higher levels of self-reported post-operative pain following PSF than their male counterparts, both in the early postoperative period (< 24 h), as well as in the subsequent 24 h time interval. Similarly, these patients received greater number of morphine milligram equivalents, despite receiving similar milligrams of the only scheduled postoperative pain medication, acetaminophen, during the postoperative course (2811.6 vs 2762.5, t = 0.2, df = 135, p = 0.85), as well as similar intraoperative pain management regimen with respect to all intraoperative medications (Table 2). However, when evaluating other outcome measures, the increased degree of postoperative pain and opioid use did not result in significant differences in time until first ambulation or time until hospital discharge. Previous literature suggests that, in addition to postoperative pain scores, factors including patient ethnicity (non-Hispanic > Hispanic), length of surgery, and number of levels fused are associated with a prolonged postoperative length of stay following PSF for AIS [25]. There were no significant differences in any of these factors between the female and male patients in this study, potentially contributing toward the similar hospital length of stay.

## Conclusion

The reduction of postoperative opioid use functions to limit adverse outcomes such as increased risk for persistent opioid use and overdose. In the adolescent population, there are a number of biopsychosocial factors that influence pain perception. This study suggests that adolescent females receiving posterior spinal fusion for adolescent idiopathic scoliosis may experience greater levels of postoperative pain and may benefit from increased preoperative counseling and more aggressive intra- and postoperative pain management regimens. Though multimodal pain management regimens have proven efficacious in reduction of postoperative pain in pooled adolescents, further research into understanding the mechanisms underlying the differences in postoperative pain perception between adolescent males and females is necessary to determine appropriate avenues for further reduction of postoperative pain and concomitant opioid use.

## Data Availability

Primary data for this project are available per request from corresponding authors.
